# Perspectives on Motivation and Change in an Intervention for Men Who Use Substances and Perpetrate Intimate Partner Abuse: Findings From a Qualitative Evaluation of the Advance Intervention

**DOI:** 10.1177/0886260521997436

**Published:** 2021-03-09

**Authors:** Sandi Dheensa, Gemma Halliwell, Amy Johnson, Juliet Henderson, Beverly Love, Polly Radcliffe, Liz Gilchrist, Gail Gilchrist

**Affiliations:** 1 University of Bristol, UK; 2 University of Worcester, UK; 3 King’s College London, UK; 4 University of Edinburgh, UK

**Keywords:** intimate partner violence, intimate partner abuse, substance-related disorders, substance use, perpetrator program, intervention

## Abstract

Despite consistent evidence that substance use is a contributory risk factor for perpetration of intimate partner abuse (IPA), little evidence exists for effective interventions for male IPA perpetrators who use substances. The Advance intervention aimed to meet this need. This 16-week intervention addressed both IPA and substance use, and was for men accessing substance use treatment who had perpetrated IPA toward a female (ex-)partner within the last 12 months. Two key theories underpinned the intervention: goal theory and self-regulation theory. In this article, we aim to illustrate the views of men and substance use treatment staff on men’s motivations to change, the ways in which men and staff said that men had changed their behavior, and the aspects of the intervention that they reported were key in the process of change. Using framework analysis, we analyzed data from 12 men who took part in the intervention as well as 31 staff members from substance use treatment services. Our five overarching themes were personal goal setting and motivation; recognition of IPA and the substance using lifestyle; improved self-regulation; considering the impact on others; and learning together in a group. Men and staff valued having a program that integrated IPA and substance use and thought the program was unique and much needed. Moreover, our findings suggest that goal theory, self-regulation, and more broadly, motivational and strengths-based approaches with practice-based activities, may be beneficial for effecting change in the substance using perpetrator population. However, further research is needed to determine the effectiveness of the intervention. Overall, our findings highlight the value of using qualitative outcome measures of perpetrator programs to complement quantitative measures of impact.

## Introduction

Global prevalence of physical and/or sexual intimate partner violence among all ever-partnered women is reported as 30% (World Health Organization, 2013). Substance use, particularly alcohol use, is a contributory risk factor for perpetration. Gilchrist et al.’s (2017) cross-sectional research calculated lifetime prevalence and identified factors associated with perpetration of physical, sexual, or emotional intimate partner abuse (IPA) by men in substance use treatment in England and Brazil. At 74.6% (77.3% in England and 72.5% in Brazil), prevalence was much higher than in general population (e.g., 29% in Brazil, Fleming et al., 2015) and general practice (e.g., 16.4% in England, Hester et al., 2015) samples from these countries. Cafferky et al.’s (2018) meta-analysis examined the strength of the relationship between substance use and IPA perpetration and victimization. Data from 285 studies and a combined sample size of 627,726 showed substance use (and alcohol use/drug use alone) was significantly related to IPA perpetration with mean effect sizes ranging from .18 to .23. No significant differences were found between the impact of different drug types. Spencer and Stith’s (2020) meta-analysis of risk factors for intimate partner homicide of women by men found perpetrator substance abuse increased likelihood by 85%. Several models are proposed to explain the relationship between IPA and substance use (see Radcliffe et al., 2021)

In Gilchrist et al.’s (2017) study, few men had ever received support from perpetrator interventions. In fact, a recent systematic review of perpetrator interventions in health settings, including substance use treatment settings, showed that interventions often exclude men with substance use disorders (Tarzia et al., 2020). The Advance intervention aimed to address this need. The program was an integrated substance use and IPA intervention for men in treatment for substance use who had perpetrated abusive behaviors toward a current or female partner within the last 12 months. Following guidelines from the UK accreditation body for perpetrator programs (Respect) the program was manualized and intended to be delivered by one male and one female facilitator with substance use and IPA expertise. In this article, we present findings from a multicenter feasibility randomized controlled trial (RCT) of the Advance intervention. Specifically, we present qualitative research from the nested formative evaluation with men who took part in the intervention and staff from the substance use treatment services involved in its delivery. The research focuses on men’s motivations for taking part and aspects of the intervention that men and staff reported were beneficial to reducing substance use and IPA. Before describing Advance and presenting findings of our analysis, we turn to qualitative literature to illustrate what is already known about participant motivations for participating in perpetrator and substance use programs, and how programs contribute to change. Regarding IPA, we focus on research with male perpetrators and female victims, and where male samples are diverse, for example, in terms of ethnicity (e.g., Holtrop et al., 2017)

### Qualitative Research on IPA/Substance Use Programs: Motivations and Change Processes

Several meta-analyses (e.g., Arce et al., 2020; Arias et al., 2013; Babcock et al., 2004; Cheng et al., 2021) have indicated mixed results on the efficacy of perpetrator programs, partially attributable to methodological issues, such as effectiveness being measured in a range of ways. Given this, there have been calls to improve the way that perpetrator programs are evaluated (Akoensi et al., 2013; Hester et al., 2014) as well as calls to better understand how perpetrators change (McGinn et al., 2020) and their motivations to enact change (Forsdike et al., 2021). Qualitative research can add nuance to evaluations by illuminating motivations and change processes. While a growing number of RCTs, systematic reviews, and meta-analyses have explored the effectiveness of perpetrator interventions for people who use substances and/or motivational strategies in IPA (e.g., Murphy et al., 2018; Stephens-Lewis et al., 2021), no published qualitative studies have done so. However various qualitative studies, including a recent systematic review (McGinn et al., 2020), have provided insight on motivation in IPA interventions. Only a handful of qualitative studies has explored motivation in substance use treatment groups. We discuss these studies here because they illustrate how qualitative research can provide a valuable contribution to evaluation research. Although this literature focuses on IPA-only or substance use-only programs, the underpinning theories are similar to those underpinning Advance and so the studies provide context for understanding our findings.

Men cite a range of motivations for engaging in perpetrator programs. McGinn et al.’s (2020) review found motivation needed to be at least partly intrinsic or existential for change to happen. That is men must have wanted to change because “it was the right thing to do.” Men with existential motivation—the desire to be a better person—were more likely to sustain change (McMurran & Ward, 2010). Extrinsic motivation, that is, where men joined IPA programs to get something they wanted (e.g., “win” their partner back [Buchbinder & Eisikovits, 2008]), was particularly common among men whose attendance was court mandated. Some of these men reportedly simulated rather than truly experienced change (McGinn et al., 2020). Intrinsic and existential motivation—for example, desire to have better health and a better life, rather than extrinsic motivation—for example, wanting to avoid jail—has also been linked to substance use treatment engagement (Dillon et al., 2020; Yang et al., 2018).

Regarding processes of change, a range of qualitative studies has found that men who perpetrate violence have cognitive distortions (e.g., about relationships, responsibility, and blame), which are the products of deeply embedded core beliefs. It is argued that men who have perpetrated IPA use these cognitive distortions to minimize and justify violence (Forsdike et al., 2021; Parra-Cardona et al., 2013). Men report that perpetrator programs have led to them becoming more aware of their own cognitive distortion and core beliefs (McGinn et al., 2020; Parra-Cardona et al., 2013), although it is unclear how far being made aware of these beliefs and distortions led to change. Other programs have drawn on emotional dysregulation approaches and have reportedly led men to better recognize triggers and warning signs for perpetration (e.g., through bodily sensations) and emotions such as anger (Morrison et al., 2018). Men report that programs have helped them understand influences on and root causes of emotional dysregulation such as entrenched social norms and social learning (e.g., learning from their fathers that “boys do not cry”; Brown, 2004; Holtrop et al., 2017).

Several studies have identified activities and techniques used in perpetrator programs that appear key to the process of achieving change. For example, activities that explore with men the different types of IPA, such as coercive control, financial abuse, and verbal abuse have reportedly led men to start recognizing their own behaviors as abusive (Holtrop et al., 2017; Morrison et al., 2018; Ormston et al., 2016; Scott & Wolfe, 2000; Scott & Wolfe, 2003). Other activities include those that improve communication and listening skills (Morrison et al., 2018; Smith, 2011), develop empathy, for example, through perspective-taking, and promote self-efficacy (as opposed to lack of control) and assertiveness (as opposed to aggression or passiveness; Scott & Wolfe, 2000; Scott & Wolfe, 2003; Smith, 2011). Men report that these aspects of programs help them to acquire a range of tools for managing intense emotions and conflict in healthier ways (Holtrop et al., 2017; Morrison et al., 2018; Ormston et al., 2016). Limited equivalent research exists about substance use interventions, although Brownlee et al. (2017) found that perceptions of activities as childish, and lack of facilitator support with tasks, diminished treatment engagement.

Therapeutic alliance with program facilitators has been reported as another important factor in the change process in IPA and substance use interventions (Dillon et al., 2020; Holtrop et al., 2017; Sotskova et al., 2016) and lack of alliance a major reason for attrition (Palmer et al., 2009). Notably, from substance use research, clients and service staff have said that the substance service’s ability to meet holistic needs such as mental health, physical health, and family violence is key in engaging clients and motivating them to continue treatment (Browne et al., 2016; Dillon et al., 2020). Group cohesion and group dynamics can also be a factor in eliciting change. In IPA and substance use interventions, participants report that in groups they feel able to share experiences and treatment goals, learn from and challenge each other, self-reflect via their different perspectives, and hold each other to account (Holtrop et al., 2017; McGinn et al., 2020; Morrison et al., 2018; Sotskova et al., 2016; Woolhouse et al., 2013; Yang et al., 2018). In substance use interventions, group factors reportedly contribute to participants recognizing their substance use as a problem—a crucial factor in treatment engagement (Yang et al., 2018). Woolhouse et al. (2013) found the nonjudgemental group environment led to self-reflection, which led many to conclude that substance use was negatively affecting their health and in turn to reduce their use. In IPA interventions, group factors have reportedly led to changes such as taking responsibility for actions (Holtrop et al., 2017; McGinn et al., 2020; Morrison et al., 2018). However, group influences can also be negative: Morrison et al. interviewed 76 IPA perpetrator program participants and found they had encountered group members who were resistant to change, or who appeared to lie or deny their abuse. Men said that such participants deterred group discussions and undermined motivation to participate and change (Morrison et al., 2018). Negative/traumatic effects of hearing others’ stories, distrust of others, and disliking being in groups generally, contributes to dropout from group substance use programs (Brownlee et al., 2017).

A caveat with all the IPA studies reviewed is that they do not measure any longer-term change and rely on men’s reports of change. Programs have aimed for men to “admit” to their perpetration, take responsibility for their abusive behavior (Holtrop et al., 2017; Morrison et al., 2018; Scott & Wolfe, 2000), and be accountable to themselves and to others in the group (Pandya & Gingerich, 2002). Holtrop et al. (2017) found these changes were the most challenging parts of their intervention, and by its end, many participants were only just beginning to grapple with taking responsibility. Nevertheless, these studies succeed in providing nuanced insight into men’s reported change processes. Assessment of reported cognitive and behavioral changes in attitudes, beliefs, and behaviors underpinning IPA can serve as complementary outcome measures to evaluate perpetrator programs, alongside quantitative reductions in IPA (Morrison et al., 2018; Stephens-Lewis et al., 2021).

In this article, we aim to illustrate views on men’s motivations to change their behavior, the ways in which men themselves and substance use treatment staff said that men had changed, and the aspects of the intervention that they reported were key in men’s process of change. Since Advance was an intervention that integrated IPA and substance use, our work adds novel findings to the body of literature we have discussed. To our knowledge, this is the first qualitative study to explore men and staff experiences of such a program. It is important to note that although we relay reports from substance use treatment service staff and men, we do not rely on these reports to claim that the intervention led to behavior change.

## Method

### Advance Intervention

We designed the Advance intervention by translating evidence around IPA and substance use into an integrated program by following the steps of the Behavior Change Wheel (Michie et al., 2011). We report on this elsewhere in detail (Gilchrist et al., 2021). Our multidisciplinary research team collaboratively developed materials based on this development work, informed by a systematic review of evidence (Stephens-Lewis et al., 2021) and primary research with dyads (Love et al., 2021). We sought feedback on materials from a “learning alliance” (professionals and academics) and a public and patient involvement group.

The Advance intervention was based on voluntary attendance, and uniquely, was delivered within substance use treatment services. It comprised two to four one-to-one preparation sessions with a keyworker (a support or treatment delivering staff member at the substance service), followed by 12×two-hour group sessions (see Supplementary Table 1 for detail on sessions). Goal theory and self-regulation (Langlands et al., 2009) were key theories underpinning the program. Goal setting allowed men to tailor the intervention to their experiences and sought to build on individual motivators. Poor self-regulation has been indicative of IPA perpetration (Finkel et al., 2009) and hazardous use of substances, while higher self-regulation has been linked to longer length of abstinence as well as reduced violent inclinations (Ferrari et al., 2009; Foshee et al., 2009; Muraven et al., 2005).

Men were encouraged to identify specific, measurable, achievable, realistic, and time-limited (SMART) goals in their one-to-one sessions and revised them throughout the program. Keyworkers (i.e., the staff members tasked with providing men one-to-one pregroup sessions and between-session phone calls) and group facilitators (i.e., those who delivered the group sessions) worked with men to encourage goals that were not extrinsically motivated (e.g., contact with children, or rekindling relationships with [ex-]partners) or outside of their control (e.g., wanting [ex-]partners to change their behaviors). In the subsequent group sessions, facilitators encouraged and supported men to identify their own risks for IPA and substance use, their activating events (triggers, unhelpful thoughts, difficult feelings, and IPA/substance using behaviors), poor responses, including poor self-regulation (limited ability to manage behaviors and make prosocial choices) and maladaptive coping (e.g., including emotional coping, problem-focused rather than solution-focused coping, and avoidant coping, through substances or blaming others), and to recognize areas that needed to change. Facilitators then introduced the skills that would encourage behavior change. The program gave men the opportunity to practice self-regulation skills through group discussions and individual and group activities. Activities were drawn from cognitive behavioral therapies, distress tolerance, and strengths-based approaches, which have shown promise within IPA perpetration prevention (Bowen et al., 2019). Skills included behavioral analysis, relaxation (e.g., breathing, muscle relaxation), use of self-soothing sensory items, and behavioral risk management tools such as “time-out.” Advance highlighted the importance of using time-out as a shared crisis management tool that must not be misappropriated to continue abusive behaviors: importantly, time-out is an effective strategy for reducing IPA risk when agreed in advance with partners (Wistow et al., 2017).

We used a trauma-informed approach and incorporated an acknowledgment of trauma and its impact on substance use and IPA into every session. This trauma-informed approach included techniques and skills such as self-soothing items, relaxation, and emotional check-in and check-outs in each session. We also dedicated an entire session to the impact of abuse on children and its links to substance use and IPA in adulthood (see Supplementary Table 1). Sessions also incorporated vignettes and videos (where actors depicted IPA/substance use scenarios, based on real-life examples from our qualitative dyad research) to foster understanding and insight. Each participant received a workbook to record progress for future reflection and improve the chances of maintaining behavior change. Men received a phone call between sessions from the keyworker who delivered their one-to-one sessions to check understanding, reinforce learning, and encourage application of material and engagement in line with men’s learning needs. The program was manualized and its content was cumulative and designed to reiterate core messages. While the material was built on the previous week’s content, it was possible for men to miss a session and re-join at a later point.

### Recruitment and Participants

Ethics approval was granted by the NHS London—Fulham Research Ethics Committee (Reference: 18/LO/0492). The trial was registered (ISRCTN 79435190). We conducted the intervention in three different sites—Site 1, Site 2, and Site 3. In both Site 1 and 2, we delivered the intervention twice and refer to these as cycles (Cycle 1 and Cycle 2). In Site 3, we delivered the intervention a third time (Cycle 3) due to high attrition in Cycle 1. In total, 54 men were randomly allocated into the intervention. We intended for there to be around nine men per group; however, we experienced high rates of attrition. It was a closed group, with all participants recruited before the intervention started. Men’s attendance was noncompulsory but encouraged.

A coordinated response that supports the needs of victims/survivors while addressing the behavior of perpetrators in the substance using population may be more likely to effect prevention of IPA (Clarke & Wydall, 2013; Davies & Biddle, 2018; Diemer et al., 2013). Women’s support services thus offered tandem support to female (ex-)partners. Alongside this, there were regular case management meetings between substance use treatment and domestic abuse services to safety plan and risk assess. Risk management was thereby central to Advance.

We offered an individual interview or focus group to all men who were allocated to the intervention, including men who had dropped out, and all facilitators and keyworkers from the six substance services that delivered the intervention. We present data from semi-structured interviews/focus groups with 12 men from the intervention group: seven interviews and a focus group with five men. In terms of ethnicity, four men were white British, one white mixed (British and gypsy/Irish traveler), three black Caribbean, three South Asian, and one Middle Eastern, and they used a range of substances. Some reported that their (ex-)partners also used substances. Three men had no children; nine men had children; and those who were separated from partners were in contact with ex-partners. We sought men’s reasons for noncompletion. One man (P11) dropped out after the first session due to having childcare commitments and another (P12) after the third due to moving abroad. Of men who missed sessions, most provided no reason for nonattendance. Of those who provided a reason, P10 missed the final five sessions due to illness but did not formally drop out. Others were ill, had other appointments, or were in hospital or rehabilitation. Possibly linked to this, some men did not wish to travel. While we can make assumptions about these reasons, we cannot reliably infer that men’s nonattendance was linked to motivation. Table 1 shows the number of sessions each man attended.

We additionally present data from semi-structured interviews/focus groups with 14 facilitators and 17 keyworkers. We conducted separate interviews/focus groups with women’s support workers and two interviews with men’s (ex-)partners but do not report on that data here. Interviews and focus groups took place in a private room by experienced and trained researchers. Men received £20 for participating in an interview/focus group. Interviews were digitally recorded and transcribed verbatim.

**Table 1.** Number of Sessions Each Man Attended (/14).

Participant ID (Advance ID)	Compulsory one-to-one (2) and Group Sessions Attended (12)
Participant ID (Advance ID)	Compulsory one-to-one (2) and Group Sessions Attended (12)
P1(P010093)	6
P2 (P010095)	9
P3 (P010099)	11
P4 (P010100)	9
P5 (P010103)	9
P6 (P020017)	13
P7 (P020041)	10
P8 (P030003)	7
P9 (P030013)	13
P10 (P030024)	5
P11 (P030032)	3
P12 (P030038)	5

### Qualitative Analysis

Supplementary Table 2 shows a sample of questions asked in interviews/focus groups. We used this guide flexibly, adapting our questions and phrasing according to what participants said. We adopted framework analysis (Ritchie & Spencer, 2010), which entails five steps: familiarization; identifying a thematic framework; indexing; charting; and mapping and interpretation. This approach was useful because it enabled the delineation of themes in relation to prespecified research questions, which in this case were about the acceptability and feasibility of Advance. We systematically coded and analyzed the data using a matrix with predetermined themes as our columns and transcripts as rows. We used goal theory (personal goal setting) and self-regulation theory as our theoretical frameworks to analyze and organize data in our matrix. We broke down these larger themes into subthemes, each of which had its own column. The subthemes in the matrix related to motivations for participating as linked to men’s goals and their experiences of change regarding thinking, emotions, and self-reported behaviors. We coded each participant’s transcript according to these subthemes. As we analyzed the data, other themes emerged around relationships with facilitators and group dynamics, which we added to the matrix.

Through repeated readings of the first few transcripts (familiarization), PR and BL identified the thematic framework. SD and GH then led on indexing, charting, adapting the framework, and mapping and interpretation, and regularly discussed how to interpret the data. BL and AJ each reviewed selected transcripts to ensure SD and GH had captured all relevant aspects. We used NVivo (v12) to facilitate our analysis.

## Findings

We present five overarching themes about men and substance service staff’s experiences of the intervention, organized in terms of goal theory and self-regulation (the two key theories underpinning Advance). The themes are personal goal setting and motivation; recognition of IPA and the substance using lifestyle; self-regulation; considering the impact on others; and learning together in a group. An overarching finding is that men who dropped out or attended intermittently also reported that they gained from the sessions they had completed.

1. **Personal goal setting and motivation**

In all cycles, staff emphasized the importance of recruiting men ready and motivated to deal with their substance use and IPA:

I don’t think you could let anyone on the program just because they were abusive. You do have to have the desire to change … even a little bit (Female facilitator, Site 1, Cycle 3).

Keyworkers discussed motivation with men and guided them to decide on three goals, with at least one goal focusing on nonabusive behavior in their pregroup sessions. In their interviews, men discussed different motivations: a few focused more on their substance use than on their IPA:

I wanted to find a way to improve myself and to make me a happier person by not using [substances] (P4).

Many other men had motivations related to their relationships. Some of these were general, for example, wanting to understand recurrent patterns of behavior; feeling that “things have got to change” (P6); and “being in a new relationship … [and wanting] to address some issues I had” (P11). Other motivations were more specific, for example, “improving the quality of our communication” (P12) and “rebuilding trust between my wife and me” (P12). Facilitators, keyworkers, and men emphasized the importance of one-to-one sessions for setting personal goals, saying that the sessions enhanced motivation. One-to-one sessions provided space for men to talk about themselves and their relationships, and identify their own goals and motivators before the structured intervention began. For some men, identifying goals and motivators was a key step in acknowledging the things they needed to change in their relationships. Two men missed the opportunity to have a one-to-one session before the group started and experienced difficulties as a result:

I didn’t know what about this course, why I’m going to go there, what I’m going to hear, what I’m going to learn, I didn’t know that and then when I went there, I see (P8).

Facilitators felt SMART goals were important for maintaining motivation and attendance:

The SMART model … would … remind [men] why they are there. And that felt like a bit of a spine to it (Male facilitator, Site 1, Cycle 2).

2. **Recognition of IPA and the substance use lifestyle**

Advance developed men’s understanding of the different forms of IPA and the ways in which they had been abusive:

I’ve learnt more of how abusive I was: I had that fixed idea [that it was] shouting and throwing things. Mine was a lot more subtle and insidious almost (P9).

The thing about money resonated with me … .I had some controlling behavior revealed, which I didn’t consciously know was controlling behavior (P12).

Men were consistently asked to consider how their substance use lifestyle (i.e., craving, acquisition, and withdrawal, as well as intoxication) affected their relationships, which men said they had “never thought about … before” (P9). Video scenarios were a particularly effective mechanism for eliciting this insight. As P12 said, “I could see exactly what I was doing, I could see myself there.” Staff also felt that video scenarios were effective:

The videos were good … it showed you the bloke’s point of view. Then … the woman’s point of view of what was actually happening. That’s when it made them stop and think about what they were doing (Female facilitator, Site 2, Cycle 1).

Substance service staff enthused about integrating IPA into their work and saw Advance as “a great opportunity for people to look at” IPA (Male facilitator, London Cycle 2). Men thought the integration worked well, with Advance building on skills learnt in substance use treatment for relapse prevention, for example, time-outs and crisis planning. Men talked about how they had used their time-out (e.g., “I take the dog out and I just cool down”, P10) and crisis plans. The integration of IPA and substance use was evident in men’s comments about how Advance—in the following case, alongside partner support—had contributed to reduced substance using behaviors:

There are days, now, without drinking. That was a shock because I wasn’t like that before. I’d always have to drink because I’d be ill with shakes. Now I can go two or three days without it. I haven’t got to have it. It’s my brain telling me, “Get a drink tonight.” My girlfriend helps me. She says, “You don’t want to go back.”

Interviewer: What’s helped you reduce your drinking?

The videos and stuff like that (P10).

3. **Self-regulation**

The intervention encouraged men to improve self-regulation using various tools learnt throughout the sessions. We organize this theme under three subthemes relating to triggers, thoughts, and core beliefs; coping with distress and jealousy; and learning healthier communication behaviors.

a. Recognizing triggers, automatic thoughts, core beliefs, and responses

The first stage of self-regulation in the Advance framework was for men to think about how intoxication, acquiring substances, withdrawal, and craving affected the triggers that led to automatic thoughts, feelings, and behaviors. Men reported that this process helped them to identify triggers and process their emotions in a healthier way. For example, one man said he would now openly discuss potential triggers with his wife. Another man talked about recognizing his partner’s friendship with a man as a would-be trigger and challenging the subsequent assumptions—she was “fancying someone else or cheating.” He learnt from Advance to instead “go on facts” and to look at things from “other perspectives”:

It’s understanding the person in the relationship. The stuff I was arguing about could have been because of my own interpretation of it, [which] could be clouded because of the issues that I bring, what I went through. Paranoid thinking. So if I’m thinking my partner is interested in someone else, that could just be my insecurities. There’s a lot of stuff that I picked up [from the session] … it’s taking a situation and thinking more about it, rather than doing something without thinking (P11).

Advance aimed to push men to challenge their thoughts, including automatic thoughts, by identifying and challenging the core beliefs underpinning them. Men initially identified core beliefs around what it means to be a man, and in a later session, core beliefs about relationships more generally. They explored how these beliefs can lead to abusive behaviors and ways to challenge them more critically. One man discussed beliefs underpinning his use of financial abuse:

I would always have a fear of, “If I don’t provide what I think the man should be doing,” whatever that is, working enough, having enough money, being able to afford a decent home for the family, “I will get dumped.” I believed that if you don’t [provide], women want to go to a man who’s better than you—a bigger man. I was like, “Oh god, I’m in a bit of debt … a man shouldn’t be … he should have enough money.” Then, what I would then do was avoid by hiding it (P9).

Facilitators said that having structured time to talk about core beliefs—and to do so in a group setting—led men to share beliefs that would otherwise go unvoiced and unchallenged:

This guy was only young, and he said, “I don’t know if you’re going to like this but I grew up in this family, this is what my dad was like, this is what I was told about women,” and he was literally cringing as he was saying it. There was something … that allowed him to say, “Well if we’re talking about our beliefs, let me tell you mine.” And although he was a bit fearful about being judged, it gave him the connection [to share these beliefs] (Male facilitator, Site 1, Cycle 2).

As well as identifying and challenging triggers, automatic thoughts, and core beliefs, Advance facilitators asked men to identify the costs and payoffs of IPA and substance use as a form of functional analysis. Men first identified costs and payoffs (i.e., benefits) for themselves, then costs for their (ex-)partners. A few men found this aspect especially useful:

[The most useful thing was looking at] the payoff when we do things like these. What’s a positive thing for us and then what’s negative things for us and … our relationship, how we can damage our relationship … how we should be, we should act, all those things were interesting (P8).

Crucially, Advance emphasized that IPA provides no payoffs for the (ex-)partner, highlighting its negative impact on them. Advance asked men to look at how short-term gains or payoffs for themselves had kept behavior going, even with high costs to themselves and their (ex-)partners, and ultimately prevented them from achieving long-term healthy relationship and substance use goals. Advance encouraged men to reduce these payoffs or find healthier, ways to manage their behavior.

b. Learning to cope with distress and jealousy

Advance encouraged men to become more aware of their feelings and their antecedents. In turn, it encouraged them to be less reactive in situations that they would ordinarily experience as triggers to substance use, IPA, or conflict. Specifically, men found that strategies for managing distress (e.g., breathing exercises and muscle relaxation) that Advance taught to be beneficial. This was especially so as some men related their IPA and substance using behaviors in part to “find[ing] it very hard to relax” (P3). Facilitators said that men frequently talked about the impact of relaxation:

F: It was really easy for them to relate to and to use.

M: With regard to the meditation, several of them came in during the course of the 12 weeks and said, “I was going to have a row with my wife or partner today and I walked away and just meditated.”

F: “I just breathed” (Female and male facilitators, Site 3, Cycle 3).

Men said they implemented such strategies—that is, taking time to breathe and regulate emotions, in attempts to be more considered in any discussions with or responses to their (ex-)partners and others:

I started doing some breathing techniques that we learnt here that night and I did it for the first three nights. At first, I was just doing it just before I was going to bed… .When I heard something or somebody said something to me that normally I would react to straightaway, instead I was taking deep breaths and then giving a really good response about it afterwards… . Now, in every situation where I have to make a decision, I am actually taking the time to relax myself and give a firm and positive answer or response (P4).

Time outs also helped, according to men:

We still have disagreements sometimes but the length of the disagreements is not that long because I don’t … argue for the sake of arguing. I take time out for relaxation. I’ll go out for a walk or think more positively (P3).

According to keyworkers and facilitators, self-soothing sensory items were likewise beneficial and useful to implement alongside goal setting, given the course’s difficult content:

He had this thing that smelled really nicely, he’d have it in his pocket … .So it just made sense to him to draw that out … if you get to that [high stress] place. [It was] a really good idea to have a set goal and something to soothe yourself with right at the beginning because it’s heavy stuff (Keyworker, Site 3, Cycle 2).

As well as the suggested strategies for managing distress, men developed their own safety strategies for tackling negative emotions such as jealousy. For example, one facilitator described how a man in a long-distance relationship would take notice if he started to feel jealous while intoxicated or withdrawing, and would then leave his phone in a part of his home that was not easily accessible to stop himself from harassing his partner via phone.

As indicated, according to men, improving self-regulation of emotions led to them being less reactive. Advance also encouraged men to shift their attention to things within their control (e.g., their own actions) and away from things outside of their control (e.g., others’ behaviors). Men said that these positive strategies spilled over into relationships with friends and family:

P5: I wasn’t really confident … in listening to someone else say something about me and not react. I’m [still] quite reactive but now I’ve realized that someone else’s actions should not affect my actions. I can’t put a blame on someone else’s behavior for my stupidity or my actions.

Interviewer: What made you realize that or feel like that?

P5: Basically, it was some of the videos and things that I’ve heard in the past as well when people have talked about their situations.

As the quote indicates, men, as well as facilitators and keyworkers, said that Advance built on men’s pre-existing knowledge from substance use treatment of behavioral management and coping strategies. The unique value of Advance was its emphasis on *practicing* these strategies and putting them in the context of intimate relationships *and* substance use:

A lot of these things I knew already, but it was about refreshing the stuff I knew about using the coping mechanisms … and actually put[ting] them to use… Sometimes, my problem is that I’ll learn something and not put it into practice and then think, “I don’t know anything,” but now whatever I learn, I try to put it into practice. That’s one thing I’ve learnt here (P5).

As indicated, men felt that opportunity to practice led to an increased sense of self-efficacy.

3. Learning communication skills

As previous subthemes outline, men and staff valued the opportunity Advance provided the men to better understand how to communicate respectfully and clearly within their intimate relationships and with ex-partners, and to practice such communication in the embedded activities. One task, which encouraged men to understand different perspectives and that they are not always right, stood out to facilitators:

They had a picture: one way, it was a rabbit, and the other, if you turned it around, was a man with a moustache. That worked really, really well. Just getting people to go, “Okay, actually, things aren’t always what they seem” [and] relating that to interactions with their partners. (Female facilitator, Site 2, Cycle 1).

Men said that such practice benefitted their relationship. Indeed, they reported that taking their (ex-)partner’s perspective into consideration was one way in which they reappraised situations they would usually perceive as triggers for IPA and/or substance use.

A drawing task similarly gave men a chance to practice communication. One man had a “powerful” realization about the discrepancy between what he thought he was communicating and what a fellow group member had understood from his instructions:

In my eyes, I gave you the perfect information. I said, “Do a triangle at the top. Do a square at the bottom and a couple of rectangles at each side.” You did that but then when you looked at it, it was completely upside down. That opened my eyes about how important it is how you communicate what you’re trying to say to somebody. I thought that was powerful (P4).

Such tasks reportedly led some men to understand how communication is often imbued with ambiguity (e.g., P5: “I didn’t realize how loose my communication was”) and that trying to communicate clearly and respectfully is important:

A lot of the problems in relationships start from miscommunication. I can’t stress the importance of it. I can’t mention enough the value of communicating clearly and not just assuming someone has understood your point of view. I’m not saying you should be forceful and say, “Have you got it? Have you got it?” (P5).

Some men said they were now more aware of when they were not communicating clearly with their (ex-)partners, and at the same time, felt less frustrated when they felt that communication with (ex-)partners was not going well.

4. **Considering the impact on others**

After the first few sessions of Advance, men were asked to start reflecting on and considering the impact their behavior had on their (ex-)partners’ as well as other people’s (e.g., family members, children) feelings. Video scenarios and vignettes, which presented women’s experiences and perspectives of IPA and (ex-)partners’ substance use, were a key mechanism for guiding men toward this reflection, for example, recognizing emotional abuse as IPA.

[My ex-partner and I] sometimes talk to each other: she’s my friend. I can talk to her, actually, about the relationship things … how I was hurting her, because sometimes I didn’t know. I saw it in those movies … they were doing similar things and [we discussed] how it can affect the partner (P8).

Therefore, as well as managing negative feelings such as distress and jealousy in healthier ways as outlined in previous themes, men commented that they were better able to consider their (ex-)partner’s feelings. A few men reported that this consideration led them to apologize and express their gratitude toward (ex-)partners:

I’ve learnt to respect myself and the people around me and learnt to be aware of when I might have hurt somebody’s feelings and just to own it and apologize. That’s made a massive difference (P4).

I phoned my partner and said thank you for being the mother she is to our children. I don’t think I’d expressed that before… .Sitting in these sessions gave me the confidence. It wasn’t even confidence. It gave me the awareness to let her know (P1).

Considering the impact of IPA and substance use on children’s feelings and experiences was a crucial aspect of men’s experience on Advance. One session aimed to get men to recognize the impact of their own childhood experiences; identify the impact of exposure to IPA and parental substance use on children; and to develop strategies to not repeat past behaviors. This session was significant since many men were motivated to join Advance by a goal to have better relationships with their children. The session comprised of videos and the “chair exercise” based on Gestalt therapy (Paivio & Greenberg, 1995). Facilitators placed an empty chair in the centre of the room to represent a child (themselves as a child or their own children) affected by their substance use and/or IPA. Men were asked to speak to the chair: to acknowledge their wrongdoing, the child’s feelings and right not to forgive, and the harms they and the abuse had inflicted. Facilitators said that this exercise and session was:

Really powerful. In other perpetrator work I’ve done, impact on children is always an impactful one in terms of cultivating motivation to change (Male facilitator, Site 3, Cycle 2).

Fellow group members supported men while reinforcing the program’s key messages by challenging beliefs:

I always thought I had a fairly good relationship with my children but then I had an incident where I was adamant that I was in the right and she [daughter] was adamant that she was in the right. I expressed it to the group, and they showed me that maybe I wasn’t in the right and I had to go back and accept responsibility for it. I mean I accepted I’d done wrong, but I held on to that (P1).

Men without children similarly gained insight from this session. Many had been exposed to IPA and substance use in their own childhoods and this session allowed men to recognize the impact of their childhood experiences on their current behaviors. Some men claimed that they had changed their substance use and treatment of (ex-)partners as a result:

I used to think I’d never grow up drinking and being aggressive. I did grow up like my old man, do you know what I mean? It’s a shocker, to learn that. I think that’s probably why I’ve calmed down a bit as well.

Interviewer. What was that session like for you?

Pretty hard to take in, but [facilitator] said, “If you want five minutes, come and chat to me (P10).

As indicated, support from facilitators in this session was essential: they offered men extra support (via a phone call) after this session due to the risk of retraumatization from their own adverse childhood experiences (Bernstein, 2000). Through the session and this support, men were reportedly able to reflect on and gain new insights into their behaviors and achieve a greater awareness relating to their children:

I’m much more aware now of what sorts of things can leave a lasting impression on the [kids]. I’m more careful of even the language I use around them. It’s little, subtle things and things that I knew already but just I’ve just freshened up on it (P5).

5. **Learning together in a group**

According to men, facilitators were a fundamental aspect of their positive experience of Advance. They made the men feel welcome and comfortable, and were enthusiastic, nonjudgmental, and supportive, despite the challenging content:

They are accommodating, approachable, not in the slightest derogatory or prejudicial. We were led very sensitively through that path of very hot coals (P6).

They were brilliant … easy to speak to. Whatever you needed, they’d help you in any way to get what you wanted out of it. Or even anything going on in the moment at home or anything like that. They’d pull 10 minutes out … and speak to you (P10).

When we spoke, [facilitator] would give her attention even to a matter that wasn’t related to the group, but it was a personal matter (P3).

As the quote indicates, facilitators “led” men through Advance, guiding them to “get what they wanted” from it, that is, personal goals. Men moreover highlighted the importance of facilitators working well together:

It’s about having two really strong people … the most important thing from my point of view is … them just being very together and knowledgeable (P12).

It was good that it was run by a female and specifically [Facilitator] was outstanding (P3).

At the start of the intervention, men and staff collectively agreed upon a group agreement, with items such as being respectful toward each other. Interactions and relationships with group members were essential parts of Advance. Indeed, in Cycle 1 (and Cycle 2, the participants for which we did not interview), attendance was poor, which meant a valuable aspect of Advance was missing: as one man said, “you don’t tend to get other opinions, it’s all my opinion” (P9). Facilitators pointed out that the group format worked well because of men’s “honesty” (Male facilitator, Site 1, Cycle 2), and because men were “aware of themselves [and]… respectful” (Female facilitator, Site 1, Cycle 2), and willing to have open discussions. Men felt that the group context was useful for breaking down the stigma around perpetrating IPA. They reported that they could learn from others about changing their behaviors by reflecting on whether other group members insights may apply in their own lives. Moreover, group members persuaded each other to change and challenged each other’s problematic opinions and behaviors:

You’ve got other people who are from different walks of life from yourself, but they’re going through similar sort of things. They were helpful (P11).

The group persuaded me of the error of my ways. It’s better. I needed to try and open up and be more honest with my partner and it worked for me (P1).

I’ve learnt so many things when I participated in this group … I heard the different opinions of different people, there was a lot about my attitude toward the relationship with my wife (P2).

Men felt that other group members were well placed to challenge them since their situations were similar.

## Discussion

This study presents qualitative research from the nested formative evaluation of a randomized controlled feasibility trial of the Advance intervention. We have illustrated views on men’s motivations to change, the ways in which men and staff said that men had changed their behavior, and the aspects of the intervention that they reported were key in their process of change. Participants noted improvements in recognizing IPA and its interaction with the substance use lifestyle, regulation of emotion, coping with distress, and communication with others. They valued having a program that integrated IPA and substance use and felt that such a program was unique and much needed. Our findings suggest that personal goal setting, self-regulation, and more broadly, motivational and strengths-based approaches in programs, may be beneficial for effecting change in this population.

In terms of personal goal setting, men identified several goals related to relationships and substance use, demonstrating their motivation to change. Our research lends weight to the finding that intrinsic motivations are key to change (McGinn et al., 2020). Staff highlighted the importance of personalized goals set within individual and group sessions as a means of enhancing motivation and aiding retention. While engagement with goals is more likely when set by the individual rather than other people (Ryan & Deci, 2000), staff worked collaboratively with men to help shape goals and enhance motivation for behavior change.

Men’s comments also reflected the benefits of the self-regulation model for managing substance use and the risk of IPA behaviors. The finding suggested that behavioral analysis of triggers, thoughts, feelings, behaviors, and consequences (costs/payoffs) combined with the opportunity to practice skills worked well. Men talked about the ability to recognize their own triggers for substance use or IPA and to see relationship conflict from multiple perspectives (particularly their [ex-]partners). Our findings lend support to existing studies that highlight that cognitive behavioral skills, imparted here within the self-regulation model, may reduce the risk of IPA among men who use substances (Easton et al., 2018; Kraanen et al., 2013; McGinn et al., 2020). Men’s comments highlighted that they had broadened their understanding of what IPA is (e.g., not just physical). As per previous research (Holtrop et al., 2017; Morrison et al., 2018; Scott & Wolfe, 2000; Scott & Wolfe, 2003), this awareness raising was a critical first step in men accepting responsibility for their actions. However, we would agree with McGinn et al. (2020, p. 105) who argue that “therapeutic work which targets emotions is complex and skilled work,” and that it is important to better understand how the effects of this type of work affects perpetrators’ (ex-)partners and families.

Specific parts of the intervention that were especially central to men’s change processes included video scenarios, sessions on gender and childhood trauma, and practice-based activities. Videos encouraged men to consider the impact of their behaviors on (ex-)partners. Earlier perpetrator work has also found perspective-taking to be a key aspect of the change process (Scott & Wolfe, 2000; Scott & Wolfe, 2003). Participants said videos were “lightbulb moments” (Tarzia et al., 2020), that is, instances of insight and realization. The session around gender stereotypes (“being a man”), which introduced the concept of core beliefs and how they impact on thoughts, feelings, and behaviors, was considered key for encouraging men to share their experiences more openly. The session acknowledging childhood trauma and impact on children was seen by men and facilitators as a motivator for change in itself; staff noted that this session was another significant moment for men in accepting responsibility for their actions. Participants valued the in-session, practice-based activities. They felt that these tasks increased awareness of IPA and its impact. Men talked about the activities that had helped them to be less reactive and manage risk more effectively: most notably, self-soothing strategies that increased distress tolerance (e.g., muscle relaxation, breathing exercises, sensory items), the activation of such strategies within their crisis plans, and communication skills. Our findings suggest that activity-based group learning may be useful for promoting change and enhancing engagement (e.g., Sotskova et al., 2016; Woolhouse et al., 2013; Yang et al., 2018) and mirrors earlier research that teaching skills around awareness of emotions (Chovanec, 2009), communication (Scott & Wolfe, 2000; Scott & Wolfe, 2003; Smith, 2011), and self-soothing and/or relaxation can lead to reported changes in behavior (Morrison et al., 2018). Our findings also support previous research that shows that time-out may reduce the risk of physical violence (and, in our population, substance use) when men use the time to think about, reflect on, and understand actions, and where this strategy is agreed in advance with (ex-)partners (Morrison et al., 2018; Wistow et al., 2017). Further work should explore victim/survivors’ experiences of time out strategies in the substance using population.

Echoing previous research (Dillon et al., 2020; Holtrop et al., 2017; Sotskova et al., 2016) men talked about the importance of the relationships they built with facilitators and peers, which they felt were safe, supportive, and nonjudgemental. These factors were especially important in our population since men were discussing two behaviors that are stigmatizing. Interactions within the group provided men with the opportunity to challenge each other’s views and learn from facilitators as well as one another. This finding highlights the value of positive social learning and of the strengths-based and motivational approach underpinning Advance. This approach recognizes facilitators’ motivational style may assist with effectiveness of IPA perpetrator programs, especially since readiness to change is often low (Murphy & Eckhardt, 2006).

While our findings suggest that personal goal setting and self-regulation skills could lead to a decrease in IPA and substance use and so might be useful approaches in programs that address either issue, a caveat is that we have not yet measured effectiveness of the approach. Advance drew on multiple theories including cognitive behavioral theory. Renehan (2020) argues that perpetrator programs that over-rely on cognitive behavioral approaches are unlikely to provide the internal and external resources men need to stop their abusive behaviors. Programs should be needs led, informed by gender, trauma, and an intersectional understanding of behavior. We would add that it is essential that men accept responsibility for IPA behaviors. Programs should entail risk management, for which including women is key. Of eight published trials that have evaluated IPA interventions for men who use substances (Easton et al., 2007, 2018; Kistenmacher & Weiss, 2008; Kraanen et al., 2013; Mbilinyi et al., 2011; Murphy et al., 2018; Palmstierna et al., 2012; Stuart et al., 2013), only three report on outcomes for female partners (Easton et al., 2007; Kistenmacher & Weiss, 2008; Kraanen et al., 2013). Our future work will continue to provide tandem support and focus more on strategies to include victim/survivor voices.

## Strengths and Limitations

A strength of our research is that this is the first qualitative study of men’s experiences of a program that was an integrated IPA and substance use program. While McGinn et al. (2020) highlight that no authors of the 27 qualitative studies they reviewed managed to interview men who dropped out of treatment, we managed to do so. We found that men who attended intermittently reported gaining from their attendance. We add to the existing literature to show the value of qualitative research in evaluating perpetrator programs. Research such as ours can provide nuanced insight into men’s reports of change processes. Overall, our findings suggest that it is worth developing complementary qualitative outcome measures to quantitative measures of IPA such as capturing and assessing turning points, and cognitive and behavioral changes in attitudes, beliefs, and behaviors underpinning IPA and substance use. Another strength is that our work spanned different UK urban contexts and may apply to other urban high-income country settings. Although we do not aim for representativeness in qualitative research, our sample was ethnically/culturally diverse, which suggests our approach was suitable for engaging men across ethnicities/cultures.

Our research has limitations. The sample of men and staff was self-selecting: those who agreed to be interviewed were likely to be those who were more engaged with the intervention and had views about the intervention that were more positive. While staff views provided clarification and triangulation of the positive behavior changes reported, we were reliant on self-reports. We were unable therefore to determine whether men had truly made changes or had simply learnt program language—“parroting program sound bites … may be indicative of a low level of engagement with intervention and low levels of motivation to change” (McGinn et al., 2020, p. 102). Our interviews were not longitudinal, so we were unable to ascertain whether any changes were maintained. Our interviews were retrospective—and while all took place within a few weeks of the intervention ending, interviewing men and women part way through the intervention would have helped to more closely understand change as a process and to pinpoint key components of the program that lead to changes in perpetration and substance use (Morrison et al., 2018). In the program itself, attrition was high, indicating a need to do more one-to-one preparatory work with men.

## Conclusion

Our study has shown the value and need for integrated programs for substance use and IPA that are tailored to the individual risks and needs of participants. Interventions that encompass personal goal setting, self-regulation, and a strengths-based approach with practice-based activities may help to facilitate behavior change among this population. Self-regulation skills in particular that focus on functional analysis of triggers, thoughts, feelings, behaviors and consequences (costs/payoffs) alongside emotion regulation and distress tolerance may be promising options in the reduction of IPA risks and management of substance use behaviors. However, further research is now needed to determine the effectiveness of the intervention and importantly to ensure that more women’s voices are captured in this research. We hope that our findings go some way to highlighting the value of qualitative work to complement quantitative measures for evaluating perpetrator programs.

## Supplemental Material

Supplemental material for this article is available online. Click here for additional data file. Supplemental Material for Perspectives on Motivation and Change in an Intervention for Men Who Use Substances and Perpetrate Intimate Partner Abuse: Findings From a Qualitative Evaluation of the Advance Intervention by Sandi Dheensa, Gemma Halliwell, Amy Johnson, Juliet Henderson, Beverly Love, Polly Radcliffe, Liz Gilchrist, Gail Gilchrist, in Journal of Interpersonal Violence

## Authors’ Note

The first two authors (SD and GH) contributed equally.

## Author Biographies

**Sandi Dheensa**, PhD, is researcher in the Domestic Violence/Abuse and Health Research Group in Bristol Medical School, University of Bristol. She is an applied social scientist whose research focuses on the health care response to gender-based violence. She is interested in using mixed methods to evaluate interventions in high- and low-to-middle income country settings.

**Gemma Halliwell**, PhD, is researcher in the Domestic Violence/Abuse and Health Research Group in Bristol Medical School, University of Bristol. She is a mixed methods researcher with a specialist interest in domestic abuse and sexual violence. She often works in partnership with domestic abuse and sexual violence agencies to deliver nonexperimental evaluations, centered around co-production and participatory approaches.

**Amy Johnson**, MSc, is a lecturer and course leader for criminology with policing, University of Worcester. Her research focuses on offending behavior and on exploring how the criminal justice system is set up to support individuals with behavioral challenges. Amy has an interest in the development of behavior change interventions and evidence-based practice, particularly within community and healthcare settings.

**Juliet Henderson**, MSc, is a project manager in the National Addictions Centre, King’s College London. Her roles include communicating with all collaborators on the Advance project and coordinating and managing data, alongside recruitment and interviewing. Her previous research projects have focused on teenage pregnancy, young families living in socio-economically deprived areas, and mental health.

**Beverly Love**, PhD, is a researcher in the National Addictions Centre, King’s College London. Beverly completed her PhD thesis in 2017 on the cycle of relapse and recovery of Class A drug using offenders who were part of the Drug Interventions Program.

**Polly Radcliffe**, PhD, is a researcher in the National Addictions Centre, King’s College London. She has over 20 years of experience of qualitative research in health and social care that has focused on gender and identity in the context of the criminal justice system, substance use treatment, and health care services.

**Liz Gilchrist**, PhD, School of Health in Social Science, University of Edinburgh, is a forensic psychologist and program director for MSc Psychological Therapies. She is a member of the Scottish Centre for Crime and Justice Research, a recognized training provider for the Risk Management Authority, and is Chair of the Scottish Advisory Panel on Offender Rehabilitation.

**Gail Gilchrist**, PhD, National Addiction Centre, King’s College London has worked in addiction research since 1995 in the United Kingdom, Australia, and Spain. She has held several large-scale grants for research exploring substance use and its relationship with psychiatric disorders, intimate partner violence, and blood borne viruses among vulnerable groups.
