# Supplementary material for: Perspectives on Motivation and Change in an Intervention for Men Who Use Substances and Perpetrate Intimate Partner Abuse: Findings From a Qualitative Evaluation of the Advance Intervention
Source: J Interpers Violence. 2021 Mar 9;37(15-16):NP13342–72. doi: 10.1177/0886260521997436 (PMC9326801; doi:10.1177/0886260521997436)
Supplement: Supplemental material for this article is available online. [file sj-pdf-1-JIV-10.1177_0886260521997436.pdf]

1. Introduction<br>2. Managing Myself<br>3. Behaviour Analysis & Gender<br>4. Impact of Intimate Partner Abuse<br>5. Children, Parenting, Substance Use & Intimate Partner Abuse<br>6. Relating<br>7. Improving Communication<br>8. Dealing with Distress<br>9. Planning to be Better<br>10. Positive Relationships<br>11. New Futures, People's Plans & Positive Activities<br>12. Recap 'What Have we Learned'

Table 3: Advance sessions

Men

- What did you think about screening and recruitment and questionnaires to the study?
- What do you think about the involvement of your current/ex-partner?
- What did you hope to get out of taking part?
- How did you feel when you went into the intervention group?
- What did you think of the intervention as a whole? (most useful/least useful aspects)
- Did you feel that the group was a safe place to be open about your relationship? Why?
- What did you think about the individual support sessions with your keyworker?
- What changes in behaviour, if any, have you made because of taking part?
- Has your relationship with your partner changed? How? Why do you think that is?
- Has your relationship with your children changed? How? Why do you think that is?
- (If answering yes to above) How do you plan to maintain these changes? What, if anything, could help you maintain these changes?
- What, if anything, would you suggest doing differently if the intervention were repeated?

Keyworkers

- How did you find it to incorporate the individual sessions into your workload?
- What parts of the individual sessions did you feel most confident/comfortable delivering? Why?
- Were there any parts of the intervention which you found challenging to deliver? Why?
- What worked well about the individual sessions and why? What worked less well and why?
- What would you change and why?
- Do you think anything was missing? What would you add?
- To what extent do you think that the individual sessions prepared men for the group intervention?

Facilitators

- How did you find it to incorporate the intervention into your workload?
- What parts did you feel most confident/comfortable delivering? Why?
- Were there any parts you found challenging to deliver? Why?
- How did you find co-facilitating with a substance use worker/domestic violence worker?
- What worked well about the intervention and why? What worked less well and why?
- What would you change and why?
- Do you think anything was missing? What would you add?
- Do you feel those that attended were engaged?
- As you are aware not everyone recruited to the intervention attended. What do you think we could have done differently to increase attendance/retention?

Table 2: sample of interviews/focus group questions
